# Climate and Soil Microsite Conditions Determine Local Adaptation in Declining Silver Fir Forests

**DOI:** 10.3390/plants12142607

**Published:** 2023-07-10

**Authors:** Isabel García-García, Belén Méndez-Cea, Ester González de Andrés, Antonio Gazol, Raúl Sánchez-Salguero, David Manso-Martínez, Jose Luis Horreo, J. Julio Camarero, Juan Carlos Linares, Francisco Javier Gallego

**Affiliations:** 1 Departamento de Genética, Fisiología y Microbiología, Unidad de Genética, Facultad de CC Biológicas, Universidad Complutense de Madrid, 28040 Madrid, Spain; 2 Instituto Pirenaico de Ecología (IPE-CSIC), 50059 Zaragoza, Spain; 3 Departamento de Sistemas Físicos, Químicos y Naturales, Universidad Pablo de Olavide, 41013 Sevilla, Spain

**Keywords:** *Abies alba*, climate warming, forest die-off, ddRADseq, SNPs, dendroecology, soil nutrients, soil microbiome, PLFAs

## Abstract

Ongoing climatic change is threatening the survival of drought-sensitive tree species, such as silver fir (*Abies alba*). Drought-induced dieback had been previously explored in this conifer, although the role played by tree-level genetic diversity and its relationship with growth patterns and soil microsite conditions remained elusive. We used double digest restriction-site-associated DNA sequencing (ddRADseq) to describe different genetic characteristics of five silver fir forests in the Spanish Pyrenees, including declining and non-declining trees. Single nucleotide polymorphisms (SNPs) were used to investigate the relationships between genetics, dieback, intraspecific trait variation (functional dendrophenotypic traits and leaf traits), local bioclimatic conditions, and rhizosphere soil properties. While there were no noticeable genetic differences between declining and non-declining trees, genome–environment associations with selection signatures were abundant, suggesting a strong influence of climate, soil physicochemical properties, and soil microbial diversity on local adaptation. These results provide novel insights into how genetics and diverse environmental factors are interrelated and highlight the need to incorporate genetic data into silver fir forest dieback studies to gain a better understanding of local adaptation.

## 1. Introduction

Climate-change-induced disturbances are increasing in frequency and severity, threatening the persistence of numerous tree species, as well as the diversity and dynamics of forest ecosystems worldwide [1]. Together with variations in the average value of climatic variables, forests will also have to deal with the risks posed by the increase in adverse weather events, broadly represented by extreme droughts and heat waves [2,3].

Tree populations can follow two main strategies to cope with major changes in climatic conditions: migration and adaptation [4]. Migration rates will likely be surpassed by the current pace of climate change [5] and could be hindered by natural barriers and human-induced habitat transformations [6]. On the other hand, the genetic diversity of populations could be key to mitigating the effects of climate change [7] and achieving adaptation through the selection of potentially beneficial alleles present in the populations [8]. Therefore, understanding the relationship between genomes and adaptive phenotypic characteristics and the extent to which they are influenced by the environment may be essential for predicting the fate of tree species coping with climate change [9,10].

Regarding genomic information, single nucleotide polymorphism (SNP) genotyping allows one to make extensive use of genetic markers, as they are found at a high frequency across genomes [11]. Genotyping by sequencing (GBS) methods for SNP discovery are particularly useful to study the genetic structures of natural populations [12,13], identify genome–environment associations [14,15], and detect selection signatures [16,17]. In addition, they are particularly appropriate for conifer genomes, allowing for a reduction in their intrinsic complexity derived from their large sizes and high percentage of repetitions [18] by reducing the sequenced portion. More specifically, double digest restriction-site-associated DNA sequencing (ddRADseq) including methylation-sensitive enzymes in the digestion step seems to be especially beneficial [14], as demonstrated by the increasing number of studies following this approach, e.g., [15,19,20]. Repetitive DNA sequences and transposable elements are highly methylated in plants [21], so the use of at least one methylation-sensible enzyme is conducive to a reduction in their representation and to an increase in the coverage of coding regions [22].

The combination of genetic data with environmental factors and dendrochronology can help provide a comprehensive vision of how genomes are shaped by their interactions with the environment, and how much of an impact they make on functional traits and growth patterns [23].

Genome–environment associations look for statistical associations between allele frequencies and ecological variations with the aim of identifying genes involved in local adaptation [24]. With climate change notably altering the abiotic environmental factors that are known to affect plants [25], the need to understand the molecular basis of such evolutionary processes is particularly important in order to be able to design conservation plans if necessary to protect plant species.

While genome–environment associations can help identify putatively adaptive *loci* [26], genotype–phenotype associations give cause for other approaches in the path to understanding adaptation. Particularly, dendrophenotypes offer an exceptional opportunity to determine how each tree responded to documented stress periods in the past by analyzing past growth rates, and have become a potent resource for genetic association studies [27]. Leaf functional traits can also add relevant information regarding responses to drought events. For instance, high leaf mass area seems to lead to better tolerance to dehydration [28], just like leaf dry matter content is positively correlated with endurance to dry conditions [29,30].

In this context, this study focuses on silver fir (*Abies alba* Mill.), which is among the tree species affected by extensive dieback and increased mortality rates [31,32]. Forest decline in populations of silver fir has been reported since the 1980s in the Pyrenees (Europe), coinciding with a succession of dry years [33]. Although it was generally agreed that this conifer is quite resistant to drought [34,35], water stress has been linked to growth decline [36], crown defoliation [37], nutritional impairments in carbon–water balance [38], and uncoupled patterns of seed dispersal and regeneration [39] in Pyrenean silver fir forests in recent decades. Predictions point to more frequent and intense droughts in the Mediterranean area [40], concurrent to global warming [41], which will further challenge the species’ ability to cope with the new environmental conditions.

Consequently, the main objective of this study was to identify molecular markers (SNPs) in the *A. alba* genome that could be used to characterize silver fir populations in the Pyrenees and contain enlightening information regarding their potential adaptive capacity to climate change. Hence, we combined genotype information with environmental data, soil physicochemical properties and soil microbial diversity, and functional and dendrophenotypic traits with the following aims: (i) to describe and compare the genetic characteristics of declining and non-declining stands, as well as declining and non-declining trees, (ii) to detect genomic regions under selection in such trees, and (iii) to identify genetic markers (SNPs) associated with environmental and phenotypic variables potentially involved in responses to climate stress. This information could help gain a better insight into this conifer’s declining process.

## 2. Results

### 2.1. SNP Genotyping

After trimming, an average of 96.87% of the reads were retained (93.54% minimum, 98.37% maximum). An average of 68.74% of those reads were successfully mapped to the reference genome (37.42% minimum, 85.19% maximum). A total of 55,803 SNPs were identified in the 266 sequenced samples, of which 3958 SNPs and 254 individuals were retained after the filtering step (Table S1).

### 2.2. Genetic Characteristics of Populations

The SNP dataset showed a percentage of polymorphic *loci* of 99.44, 88.53, 99.62, 99.72, and 98.79 in the five studied stands (CO, GA, PE, PM, and SO, respectively; see Section 4.1 for details). Significant genetic differences, measured by fixation index (F_ST_), were detected between all population pairs (Table 1). The analysis of molecular variance (AMOVA) also showed significant genetic differences among populations (Table S2). When accounting for F_ST_ differences between declining and non-declining individuals, no statistical differences were found within any population (Table 2).

**Figure 1:**
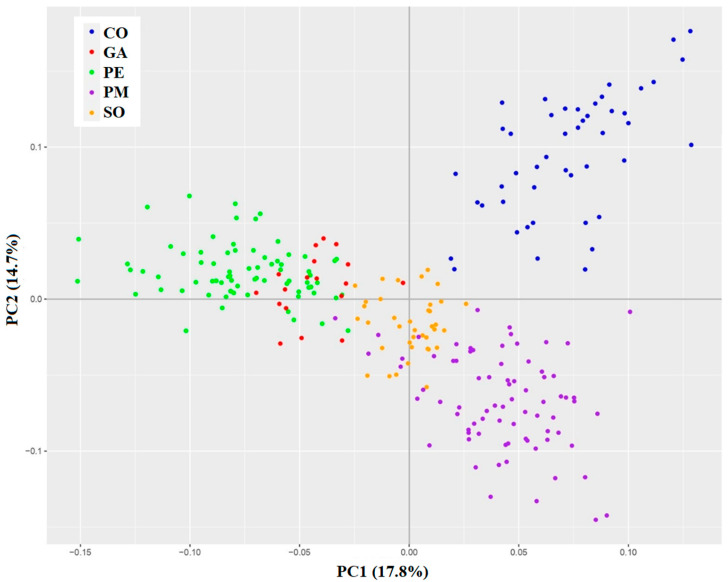
Principal component analysis (PCA) of the 3958 *A. alba* SNPs showing the first and second principal components. Each color represents a different population (CO, GA, PE, PM, SO).

The principal component analysis (PCA) showed, in agreement with the F_ST_ and AMOVA results, that each population can be genetically distinguished, with their respective individuals grouping together (Figure 1). CO was the most isolated one, in consonance with a larger geographic distance between itself and the other study sites (see Section 4.1). The two healthy populations (GA and SO) appeared close to each other, in the center of the plot. GA overlapped with PE, while SO partially overlapped with PM. This pairing was in line with the existence of geographical barriers between the two populations more to the west and the two more to the east (GA and SO are separated by a mountain, and PE and PM are on different sides of a valley).

### 2.3. Selection Signatures

**Figure 2:**
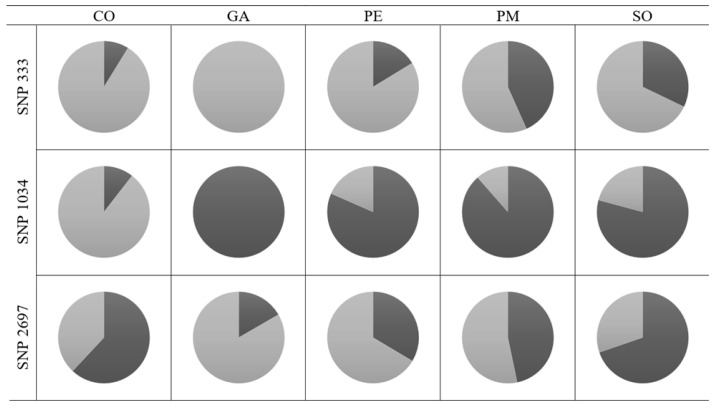
Allele frequencies of SNPs showing selection signs (SNP 333, SNP 1034, SNP 2697) in the studied *A. alba* populations (CO, GA, PE, PM, SO). Dark gray represents the frequency of allele 1; light gray represents the frequency of allele 2.

Selection signatures were found in 41 SNPs taking into account the five genetically different populations (Figure S1). The rest of the comparisons (considering health state and age) did not uncover any genomic region under selection. Eleven of those SNPs were located within an annotated gene or close to one (Table 3). Among the proteins produced by these genes, there was an abscisic acid (ABA) receptor, which is part of a family involved in responses to adaptive stresses in plants, coordinating complex networks that allow responses to drought, salinity, and temperature fluctuations [42]. A heavy metal-associated protein (HIPP) also showed signs of selection. While they are mainly known for their detoxification purposes, it has been seen that they also play a role in response to ABA, and, therefore, in response to different abiotic stress conditions [43,44]. In addition, a chaperone could also be found among the obtained proteins, which is also linked to the ABA signaling pathway. These proteins can sense the epigenetic environment created by some chromatin-remodeling factors that interact with ABA, playing a role in inducing stress memory by ensuring the dynamic epigenetic mechanisms that control gene expression [45]. Their allele frequencies in each population can be seen in Figure 2.

### 2.4. Genome–Environment Associations (GEA)

**Figure 3:**
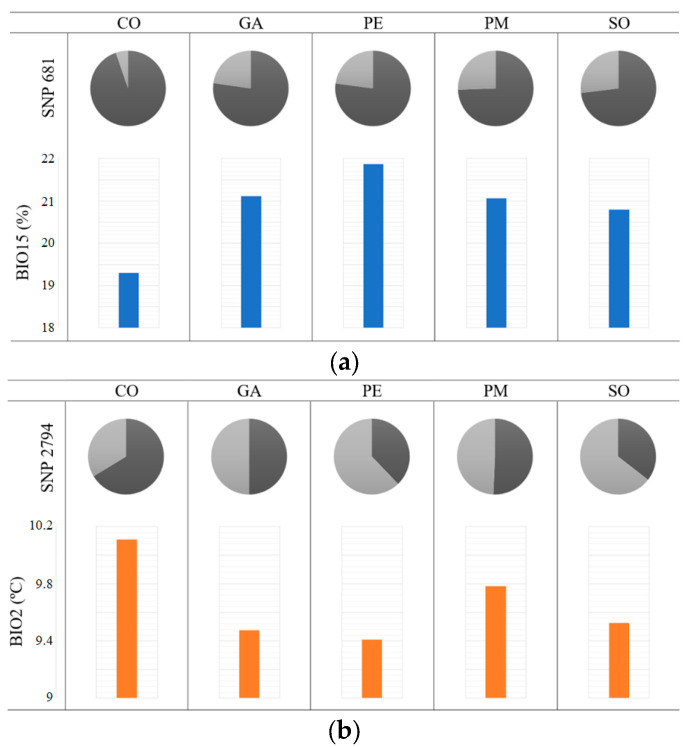
Allele frequencies of SNP 681, associated with a precipitation variable (BIO15, precipitation seasonality, represented in blue) (**a**), and SNP 2794, associated with a temperature variable (BIO2, mean diurnal range, represented in orange) (**b**). Dark gray represents the frequency of allele 1 in each population; light gray represents the frequency of allele 2 in each population.

Genome–environment associations were found between 47 different SNPs and the five bioclimatic variables, giving a total of 71 associations (Table S3). Of these, 4 also showed selection signatures, and 15 were located within an annotated gene or close to one (Table 4). BIO15 (precipitation seasonality) showed the highest number of associations, while BIO3 (isothermality) only had one. Among the proteins associated with precipitation variables, there was an aquaporin, which is part of a family of water channels crucial for maintaining water balance in plants [46]. There was an SNP in one of the introns which are part of the gene that encodes this protein, with the frequency of its allele 2 (T) increasing as BIO15 increased (Figure 3a). Temperature variables also showed associations with proteins related to response to abiotic stress, such as RAD7, which is involved in DNA repair after UV light damage [47]. In this case, the frequency of allele 1 (T) of its associated SNP increased as BIO2 (mean diurnal range) increased (Figure 3b).

Genome–environment associations were found between 35 different SNPs and the eight abiotic soil variables, giving a total of 80 associations (Table S4). Of these, 5 also showed selection signatures, and 13 were located within an annotated gene or close to one (Table 5). The Saxton value showed the highest number of associations, while the percentage of silt only had two.

Regarding the eight biotic soil variables, 33 different SNPs were significantly associated with them, giving a total of 81 associations (Table S5). Of these, 4 also showed selection signatures, and 13 were located within an annotated gene or close to one (Table 6). The concentration of Gram-positive bacteria showed the highest number of associations, while fungi only had one.

**Figure 4:**
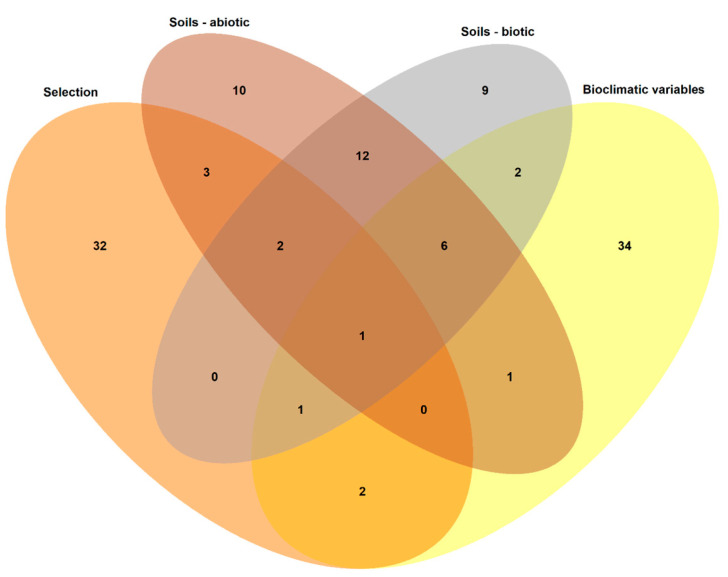
Venn diagram showing the number of SNPs associated with different environmental variables (bioclimatic variables, soils—abiotic, soils—biotic) or showing selection signs, and the coincidences among them.

A high proportion of SNPs were found to be associated with several types of environmental variables and/or to be under selective pressure (Figure 4), showing the influence of the environment on this species genome and the existence of a correlation between soil and climatic variables.

### 2.5. Genotype–Phenotype Associations (GPA)

Genotype–phenotype associations were found between 19 SNPs and 2 functional traits (out of 5) and 7 dendrophenotypic traits (out of 46) related to response to past drought events, with resilience to the 1998 drought (Rs98) as the variable with the highest number of associations (Table S6). Of these, eight were located within an annotated gene or close to one, and none of them showed either signatures of selection or associations with environmental variables (Table 7).

**Figure 5:**
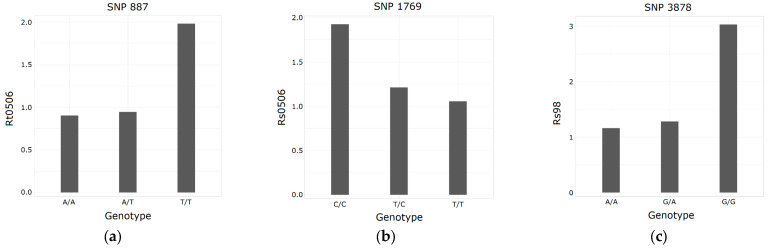
Mean values of resistance to the 2005–2006 drought (Rt0506), resilience to the 2005–2006 drought (Rs0506), and response to the 1998 drought (Rs98) with respect to genotypes (allele 1/allele 2) of SNP 887 (**a**), SNP 1769 (**b**), and SNP 3878 (**c**), respectively.

Regarding the 2005–2006 drought, there were two proteins with functions involved in drought response associated with resistance and resilience to this extreme climatic period. One of them was a zinc finger transcription factor which has been linked to drought and heat response multiple times, e.g., [48,49,50]. Homozygous individuals for the T allele of its associated SNP had a higher resistance to the 2005–2006 drought (Figure 5a). The other one was an expansin, which contributes to drought-resistance by maintaining cell turgor and taking part in ABA-mediated cell-wall extension [51]. Homozygous individuals for the C allele of its associated SNP showed a higher resilience to the 2005–2006 drought (Figure 5b).

Also associated with resilience, but to the 1998 drought period, there was an aluminum-activated malate transporter, which influences the cellular gamma-aminobutyric acid (GABA) concentration [52]. This amino acid is rapidly accumulated in response to multiple stresses, including drought [53]. Homozygous individuals for the G allele of its associated SNP had a higher resilience to the 1998 drought (Figure 5c).

## 3. Discussion

This study aimed to gain a better understanding of how genetics could be involved in tree decline and mortality, determining local variability in healthy trees and neighboring individuals showing a declining process towards death. Furthermore, we provide information about the genetic characteristics of contrasting Pyrenean populations of silver fir and their relationship with the environment and phenotypic traits, finding climate and soil microsite conditions as the main factors affecting their local adaptation.

Previously, microsatellites were the most widely employed method to study genetic diversity, structure, and adaptation in silver fir populations, e.g., [54,55,56]. More recently, SNPs have gained popularity as molecular markers in this species, predominantly in the form of genotyping a predefined list of them in candidate genes, e.g., [27,57,58]. In spite of its robustness, it is a limited approach, not accounting for genetic variability in non-coding regions. Additionally, its potential for adaptation in forest trees could rely on quantitative trait *loci* (QTL), which tend to be scattered across the genome [4], rather than just specific genes of major effect. QTL mapping requires a large number of genetic markers to be successful [59], stressing the need for genome-wide identification of SNPs. RADseq had already proved to be a useful technique for genome-wide genotyping in *A. alba* [60], and our results show that ddRADseq can be used for that purpose too.

However, genome-wide identification of SNPs in conifers, although possible, remains a complex process hindered by the lack of reference genomes and their quality. For instance, 70% of the reads used in this study mapped to the silver fir reference genome, possibly due to the highly fragmented nature of this assembly. This means 30% of the generated information was lost. It is clear that efforts must be made to improve the existing references and sequence new species.

To our knowledge, this is the first study comparing the genetics of declining or non-declining silver fir individuals. Our results showed that there were no genetic differences between them within their respective populations. *A. alba* is known to have high phenotypic plasticity, mainly attributed to the effect of environmental factors rather than genetic variability [61]. Thus, epigenomics could be playing a more important role in the fate of each tree in populations subjected to increasingly unfavorable environmental conditions than their differences in genetic molecular markers, especially since climate change is surpassing their adaptive changes in allele frequency rates [5,18]. The lack of selection signatures between adult trees and saplings could also be indicative of a slow adaptation process happening in the studied populations which is not detectable yet and, thus, clearly slower than the ongoing climatic changes. This means this species could probably be threatened in the Pyrenees if conditions continue as they are currently.

Regarding the comparison between populations and considering that they are placed in regions with different environmental conditions, it is not surprising that several SNPs showed signs of selection. Some of these could be used as molecular markers of nearby genes that are involved in stress responses, mainly related to ABA signaling pathways. Whole-genome scans in other coniferous species have demonstrated that genes under selection are often involved in responses to abiotic stress and are rapidly evolving to enable trees to cope with such demanding conditions [62,63]. Therefore, this finding suggests that the environment is impacting the silver fir’s genome by causing different levels of abiotic stress to the populations. The fact that approximately 22% of these SNPs were associated with environmental variables supports this affirmation.

Temperature and precipitation had a similar number of associated SNPs whose allele frequencies changed between populations in line with the variable’s variation. Precipitation seasonality (BIO15) had the highest number of associations, including an aquaporin, which has been directly linked to drought stress in *A. alba* [64]. Additionally, this variable seems to drive the growth timing of populations of silver fir [65], revealing itself as a key environmental factor for this species [66]. On the other hand, the most relevant temperature variable was mean diurnal range (BIO2), which has been identified as a potential modifier of the effect of drought on tree growth, alleviating the degree of stress caused by drought events when its range is narrow [67].

Soil variables also had a high number of associations, with a large percentage of SNPs associated with both biotic and abiotic parameters. Vegetation, microbial community, and physicochemical properties of soil are closely linked to each other, interacting in a process known as plant–soil feedback [68]. It has been described that plant genotype can significantly influence microbial community composition [69], which, in turn, has an impact on soil properties, such as pH, organic matter content, and soil structure [70,71], and vice versa, with the microbial community being the one affected when there are changes in soil conditions and vegetation [72,73]. Therefore, it is no wonder that almost 50% of the associated markers appear both for soil composition and soil microorganisms and are strongly related to the sampled trees’ genotype.

In addition, it has been described that forest management affects soil microbial composition, increasing soil microbial stress [74] and affecting its resistance and resilience to drought [75,76]. For instance, CO, which has been protected by the “Ordesa and Monte Perdido” National Park since 1918 with strict conservation policies [38], presents higher biomasses of the different tested groups of microorganisms (except for fungi) than the rest of the studied stands (Table S9), which suffered from intense logging activity in the past [33]. Therefore, although climate change and drought are usually the most discussed reasons behind tree dieback, human perturbations, such as logging and selective thinning, should be considered too.

Finally, the genotype–phenotype associations were fewer and almost unique for each variable. While genome–environment associations identify general trends due to their population-level nature, phenotype data can produce much more finely tuned results, as they consider tree-level information. Thus, a strong genetic signal related to growth traits is less likely to be found [27,77,78].

In accordance with this, only 7 out of the 46 dendrophenotypic variables related to growth patterns were associated with genetic markers. More specifically, these traits define how each tree responded to past drought periods. Survival to one-time extreme events, such as extended drought periods, might not be driven as much by adaptation as by each tree’s genetic variability, which could be key to increasing its chances of carrying a variant that could help it cope with the adverse climatic situation [8]. Thus, the small number of associations found for the variables related to response to past drought events could be pointing to genome regions that were helpful in maintaining certain growth rates and vitality while dealing with a specific unfavorable event but were not particularly useful in the long run. Additionally, none of these regions showed signs of selection, proving that they are not directly involved in long-term adaptation to the environment.

Two traits related to leaf characteristics also showed a low number of associated SNPs, in line with a weak differentiation in leaf morphology between individuals [38]. Lastly, the lack of associations to growth pattern variables based on tree-level BAI might be due to the influence of the environment on growth rates rather than the effect of the genotype itself, as the succession of severe drought events in combination with increasingly higher temperatures during the last 40 years has led to growth decline in different silver fir populations across the Pyrenees [33,79].

In summary, integrative studies incorporating genetic, phenotypic, and environmental data represent a powerful tool for the uncovering adaptation mechanisms of natural tree populations, as demonstrated by the growing number of publications following this approach, e.g., [14,27,58].

In this regard, improved reference genomes for conifer species are of utmost importance in the pursuit of understanding their genetic vulnerability to climate change. In addition to allowing more efficient genome-wide identification of molecular markers and adaptive candidate *loci*, they would pave the way for the accurate identification of epigenomic marks [18]. Epigenetic mechanisms reflect to a large extent the effect of environmental factors on gene expression [80], which would complement the existing information about how these variables affect the genome. Given that our results did not identify any genomic region under selection between declining and non-declining trees, epigenomic studies should also be put in the spotlight following their potential explanatory power regarding trees’ response to climate change.

## 4. Materials and Methods

### 4.1. Study Species and Sites

**Figure 6:**
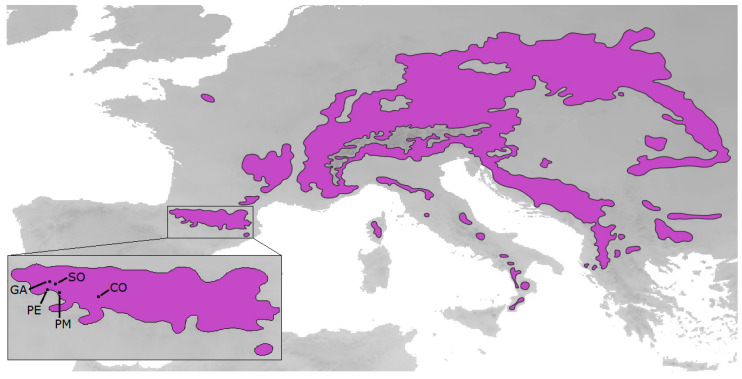
Silver fir, *Abies alba*, distribution in purple, as described by EUFORGEN, and study sites in the Pyrenees (GA, CO, PE, PM, SO), marked with black circles.

Silver fir (*Abies alba* Mill.) is a large conifer distributed in montane areas in Europe (Figure 6). It can live up to half a millennium, reaching its reproductive maturity at 30–40 years old [81]. It is commonly found in elevated areas (500–2000 m a.s.l) with 700–1800 mm of mean annual precipitation [82]. It is highly shade-tolerant, finding its best growth conditions in locations with at least 1500 mm of annual precipitation and mean temperature around 9 °C [83]. It can grow in soils with a pH from acid to neutral, preferring moist soils but not too wet [81]. The silver fir forests of the Pyrenees constitute the European southwestern limit of the distribution of this species, where it usually grows in mesic sites on north-facing slopes, creating pure or mixed forests with European beech (*Fagus sylvatica* L.) or Scots pine (*Pinus sylvestris* L.), with the understory vegetation dominated by several shrubs such as European box (*Buxus sempervirens* L.) [38].

The study sites were the four *A. alba* stands included in a previous study [38] plus an extra one, all of them placed in north-western Aragón, in the central-western Spanish Pyrenees (Figure 6, Table 8). Three of these sites show signs of forest decline, with declining and non-declining trees coexisting (Cotatuero, CO; Paco Ezpela, PE; and Paco Mayor, PM), while the other two are populated only by healthy individuals (Selva de Oza, SO; and Gamueta, GA). Samples from different types of individuals were collected at all sites: adult trees (between 40 and 220 years old, with a mean age of 111) and saplings growing nearby (no taller than 1.5 m). Additionally, adult trees were sampled regarding two additional categories at sites where it was possible: declining individuals and non-declining (healthy) individuals. Their health state was determined by their crown defoliation levels [38], considering only trees that did not show signs of any disease. This sampling design allowed us to look for differences between age cohorts and health cohorts, as well as between stands. Tree age was determined by the preformed-growth pattern found in firs [15].

### 4.2. SNP Genotyping

Fresh leaf samples were collected from 266 *A. alba* individuals. In total, 100 mg of each sample was lyophilized and used for total genomic DNA extraction with DNeasy Plant Mini Kit (Qiagen^®^, Berlin, Germany). DNA yield and purity were determined using a NanoDrop^TM^ spectrophotometer and visually inspected after electrophoresis on agarose gels. Paired-End (PE) libraries for ddRADseq [84] were constructed using *Ape*KI and *Pst*I restriction enzymes and sequenced by LGC Genomics (Berlin, Germany), with a sequencing depth of 1M. Quality of raw reads was assessed using FastQC v0.11.9 [85]. They were trimmed using fastp v0.12.4 [86] to remove low-quality bases/reads and adapter contamination. The processed reads were mapped to the *A. alba* reference genome (Abal.1_1) [87] using ipyrad v0.9.65 [88]. This reference genome had over 37 million scaffolds, with a mean scaffold length of 488.5 pb; therefore, in order to reduce the computational cost of this process while retaining its usefulness as much as possible, a shorter version of this genome was created and used as reference. It was made of the first 62,061 scaffolds, which were the longest and had annotated genetic features. The same software (ipyrad) was then used to perform an SNP calling. The obtained SNPs were filtered with VCFtools v0.1.16 [89] to retain high-quality, informative SNPs for subsequent analyses: biallelic, minor allele frequency (MAF) > 5%, maximum missingness < 50%, and only 1 SNP per 10,000 bp to remove strong linkage disequilibrium.

### 4.3. Genetic Characteristics of Populations

Different descriptive statistics were calculated. GenAlEx v6.5 [90,91] was used to calculate the percentage of polymorphic *loci* and allele frequencies of relevant SNPs in each population. Genodive v3.06 [92] was used to test population differentiation due to genetic structure (fixation indexes, F_ST_ [93]) and to perform an analysis of molecular variance (AMOVA [94,95]) with 9999 permutations. In addition, plink2 v2.00a2.3 [96] was used to carry out a principal component analysis (PCA).

### 4.4. Selection Signatures

Detection of selection signatures was performed using BayeScan 2.1 [97] with default parameters. SNPs with *q*-values < 0.05 were considered to be under selective pressure. Different configurations of groups were tested looking for significant differences in allele frequency measured by their F_ST_ coefficient: sampling points as groups, condition as groups (non-declining vs. declining), condition as groups within each population, age as groups (adult vs. young), and age as groups within each population.

### 4.5. Genome–Environment Associations (GEA)

Three types of environmental variables were considered for genome–environment associations (GEA): (1) bioclimatic variables, (2) abiotic soil variables, and (3) biotic soil variables (microbial communities). The associations were identified using BayEnv2 [98] with 100,000 iterations after estimation of the marker’s covariance matrix as indicated by the manual. SNP–variable combinations scoring a Bayes Factor (BF) > 100 were considered to be associated [99].

For each sampling point’s location, 19 bioclimatic variables were obtained from the WorldClim database [100], with a grid cell resolution of 30 s, using QGIS 3.18 (QGIS Development Team, 2022). Highly correlated variables (Pearson’s coefficient > 0.9) were removed, keeping only 5 bioclimatic variables (Table 9), of which 3 were related to temperature (BIO1, BIO2, BIO3) and 2 were related to precipitation (BIO12, BIO15).

The 8 abiotic soil variables were obtained at individual-level [38] and then their mean values were calculated for each population (Table 10). Briefly, soil samples were processed to determine percentage of N, percentage of C (organic and total, used to calculate the C/N ratio), assimilable P (ppm), and 4 soil texture variables (percentage of clay, silt, and sand, and Saxton value to integrate them in a single variable).

Finally, the 8 biotic soil variables (Table 11) were based on Phospholipid Fatty Acid (PLFA) analysis [101] to assess the microbial community found in the soils where the sampled trees lived. General fatty acid methyl esters (FAMEs) were obtained and then individually identified to calculate the biomasses of eukaryotes, Gram-negative bacteria, Gram-positive bacteria (not including actinobacteria), Actinomycetes, fungi, arbuscular mycorrhizal (AM) fungi, and anaerobic bacteria.

### 4.6. Genotype–Phenotype Associations (GPA)

Two types of tree-level phenotypic data were considered for genotype–phenotype associations (GPA): (1) functional traits related to leaf characteristics, and (2) dendrophenotypic traits related to growth patterns, climate sensitivity, and response to past drought events. Only adult trees were used for this analysis as saplings were not big enough to sample wood cores from their logs. The associations were determined using the R package rrBLUP v4.6.1 [102,103], including the 19 bioclimatic variables as fixed effects. SNP–trait combinations with *q*-values < 0.05 after FDR correction were considered to be associated.

The 4 functional traits (Table S7) included leaf area (LA, in mm^2^), which was measured while needles were fresh, and leaf mass per area (LMA, in mg mm^2^), which was calculated after they were dried [38]. Leaf dry weight (LDW, in g) was also measured, as well as leaf dry matter content (LDMC, in g g^−1^) [101].

The 46 dendrophenotypic traits were obtained from dendrochronological studies carried out using data obtained from sampled cores [38]. For the 13 dendrophenotypic traits (Table 12 and Table S8) related to tree-level growth patterns, several variables based on the basal area increment (BAI) were calculated [15], which reflect the growth of the whole tree rather than just the one-dimensional ring width. The periods 1979–1999 and 1999–2019 were selected to account for a time span prior to extensive decline symptoms in the studied *Abies alba* forests, and a near-to-present period when this fir dieback was widely reported [33].

For the 21 dendrophenotypic traits (Table 13 and Table S9) related to climate sensitivity, BAI values were correlated with different temperature and precipitation variables [15]. The period used to investigate climate sensitivity was from 1961 to 2019 to ensure the reliability of the climate data and because it included growth data from all sampled trees, excluding juvenile effects [38].

For the 12 dendrophenotypic traits (Table S10) related to tree-level response to documented drought periods, resistance (Rt), recovery (Rc), and resilience (Rs) indexes were calculated based on the ratios between different BAI values (reflecting pre-drought, drought, and post-drought growth during documented past extreme drought events) for the 1986 drought, the 1998 drought, the 2005–2006 drought, and the 2012 drought [38].

## 5. Conclusions

The sampled stands of silver fir in the central-western Spanish Pyrenees showed genetic differences among them. However, no significant genetic differentiation was obtained between declining and non-declining trees within populations. Associations between genome and environment were abundant, linking tree genetics, climate, and soil composition and microbial communities. A high percentage of them also showed signs of selection, supporting a strong impact of environmental factors on this species’ genome. Genotype–phenotype associations were scarce, indicating that growth patterns might be shaped by mechanisms beyond the tree’s genotype itself, such as phenotypic plasticity in response to extreme climatic events. All in all, we provide an integrative vision of how the silver fir genome, the environment, and its phenotype are interrelated, which could be a valuable source of information in the context of climate change.

## Figures and Tables

**Table 1:** Fixation indexes (F_ST_) above diagonal between the studied *A. alba* populations in the Pyrenees and their associated *p*-values below diagonal.

Populations	CO	GA	PE	PM	SO
CO	-	0.038	0.034	0.027	0.027
GA	0.000	-	0.028	0.035	0.034
PE	0.000	0.000	-	0.027	0.019
PM	0.000	0.000	0.000	-	0.015
SO	0.000	0.000	0.000	0.000	-
Number of samples	47	18	74	77	38

**Table 2:** Fixation indexes (F_ST_) and their associated *p*-values between non-declining (ND) and declining (D) *A. alba* individuals within each declining population (declining individuals were present only in CO, PE, and PM populations; see Section 4.1 for details).

	CO	PE	PM
F_ST_	0.004	0.004	0.002
*p*-value	0.142	0.055	0.192
Number of ND samples	15	20	19
Number of D samples	13	18	20

**Table 3:** Molecular markers (SNPs) under selection found within or close to an annotated gene (“Position”) in *A. alba*, specifying their exact location in the genome, as well as the protein encoded by such genes.

SNP	Location	Position	Protein	Protein ID
333	aalba5_s00002910:109,731	Within the gene	Abscisic acid receptor (PYL3)	AALBA5B1118705T1
799	aalba5_s00008638:65,727	Within the gene	La-related protein 1B (LARP1B)	AALBA5B999098T2
1034	aalba5_s00012107:25,977	Within the gene	Heavy metal-associated protein (HIPP39)	AALBA5B430765T1
1241	aalba5_s00014817:64,736	Within the gene	Fucosidase (FUCO1)	AALBA5B698786T1
2059	aalba5_s00027378:40,879	Within the gene	Mediator of RNA polymerase II (MD26C)	AALBA5B1091291T1
2477	aalba5_s00034336:46,604	20,152 bp downstream	Kinase (CRK42)	AALBA5B567750T1
2591	aalba5_s00036159:10,277	5615 bp upstream	Transcription factor (PHL3)	AALBA5B939227T1
2665	aalba5_s00037486:53,893	939 bp downstream	TATA-box-binding-interacting homolog (RIN1)	AALBA5B827346T1
2697	aalba5_s00038192:32,220	25,073 bp downstream	Chaperone (BAG5)	AALBA5B1063708T1
3215	aalba5_s00047689:19,730	2651 bp upstream	Kinase (BSK2)	AALBA5B446846T1
3952	aalba5_s00062077:3573	35,864 bp upstream	Squalene epoxidase (ERG1)	AALBA5B441483T1

**Table 4:** Molecular markers (SNPs) associated with bioclimatic variables found within or close to an annotated gene (“Position”) in *A. alba*, specifying their exact location in the genome, as well as the protein encoded by such genes. SNPs with selection signatures are marked in bold.

SNP	Location	Position	Protein	ID
159	aalba5_s00001283:86,646	21,883 bp upstream	UDP-glycosyltranferase (U86A2)	AALBA5B1024847T1
179	aalba5_s00001426:131,290	Within the gene	BEACH domain-containing protein (BCHC2)	AALBA5B098257T1
681	aalba5_s00007005:35,095	Within the gene	Aquaporin (SIP2-1)	AALBA5B253951T1
983	aalba5_s00011431:62,999	21,010 bp downstream	Tetratricopeptide repeat-like superfamily protein (PPR35)	AALBA5B348236T1
1808	aalba5_s00023261:31,104	Within the gene	Kinase (NEK4)	AALBA5B973686T1
2061	aalba5_s00027387:15,057	30,900 bp upstream	MOTHER of FT and TFL1 (MFT)	AALBA5B1139649T1
2119	aalba5_s00028425:12,399	328 bp upstream	Chaperone, chloroplastic (DJA7A)	AALBA5B512522T1
2459	aalba5_s00034077:43,568	772 bp upstream	Mitogen-activated kinase (M3K17)	AALBA5B244254T1
**2591**	aalba5_s00036159:10,277	5615 bp upstream	Transcription factor (PHL3)	AALBA5B939227T1
2645	aalba5_s00037229:12,665	111 bp upstream	Homeobox (PKNOX1)	AALBA5B627517T1
2794	aalba5_s00040228:24,711	20,262 bp upstream	DNA repair protein (RAD7)	AALBA5B261613T1
2940	aalba5_s00042872:51,622	18,194 bp downstream	Helicase (MAA3)	AALBA5B767119T1
3063	aalba5_s00045131:27,419	17,345 bp downstream	Phosphatase (P2C60)	AALBA5B289418T1
3313	aalba5_s00049423:3040	909 bp upstream	Translation initiation factor (SDA1)	AALBA5B666590T1
3400	aalba5_s00051396:12,444	Within the gene	Uncharacterized transmembrane protein (UGPI7)	AALBA5B360123T1

**Table 5:** Molecular markers (SNPs) associated with abiotic soil variables found within or close to an annotated gene (“Position”) in *A. alba*, specifying their exact location in the genome, as well as the protein encoded by such genes. SNPs with selection signatures are marked in bold.

SNP	Location	Position	Protein	ID
238	aalba5_s00002010:71,026	22 bp downstream	Endo-1,3(4)-beta-glucanase (ENG1)	AALBA5B495784T1
287	aalba5_s00002479:90,851	1649 bp downstream	Chaperone (DNJ10)	AALBA5B1036913T1
764	aalba5_s00008117:59,024	25,055 bp upstream	Coatomer subunit delta-2 (COPD2)	AALBA5B407046T1
1808	aalba5_s00023261:31,104	Within the gene	Kinase (NEK4)	AALBA5B973686T1
2587	aalba5_s00036112:6656	407 bp upstream	Translocase of chloroplast (TC120)	AALBA5B952204T1
**2665**	aalba5_s00037486:53,893	939 bp downstream	TATA-box-binding-interacting homolog (RIN1)	AALBA5B827346T1
2794	aalba5_s00040228:24,711	20,262 bp upstream	DNA repair protein (RAD7)	AALBA5B261613T1
3000	aalba5_s00044048:11,938	Within the gene	3-oxoacyl synthase, mitochondrial (KASM)	AALBA5B636711T1
3120	aalba5_s00046057:16,198	309 bp downstream	Sorbitol dehydrogenase (DHSO)	AALBA5B083042T1
3313	aalba5_s00049423:3040	909 bp upstream	Translation initiation factor (SDA1)	AALBA5B666590T1
3870	aalba5_s00060613:17,756	21,499 bp upstream	Ribonuclease HI (RNH)	AALBA5B308154T1
3874	aalba5_s00060695:20,797	21,154 bp upstream	UDP-glucose 4-epimerase (GALE2)	AALBA5B999424T1
**3952**	aalba5_s00062077:3573	35,864 bp upstream	Squalene epoxidase (ERG1)	AALBA5B441483T1

**Table 6:** Molecular markers (SNPs) associated with biotic soil variables found within or close to an annotated gene (“Position”) in *A. alba*, specifying their exact location in the genome, as well as the protein encoded by such genes. SNPs with selection signatures are marked in bold.

SNP	Location	Position	Protein	ID
159	aalba5_s00001283:86,646	21,883 bp upstream	UDP-glycosyltranferase (U86A2)	AALBA5B1024847T1
238	aalba5_s00002010:71,026	22 bp downstream	Endo-1,3(4)-beta-glucanase (ENG1)	AALBA5B495784T1
681	aalba5_s00007005:35,095	Within the gene	Aquaporin (SIP2-1)	AALBA5B253951T1
764	aalba5_s00008117:59,024	25,055 bp upstream	Coatomer subunit delta-2 (COPD2)	AALBA5B407046T1
983	aalba5_s00011431:62,999	21,010 bp downstream	Tetratricopeptide repeat-like superfamily protein (PPR35)	AALBA5B348236T1
1808	aalba5_s00023261:31,104	Within the gene	Kinase (NEK4)	AALBA5B973686T1
2587	aalba5_s00036112:6656	407 bp upstream	Translocase of chloroplast (TC120)	AALBA5B952204T1
**2591**	aalba5_s00036159:10,277	5615 bp upstream	Transcription factor (PHL3)	AALBA5B939227T1
**2665**	aalba5_s00037486:53,893	939 bp downstream	TATA-box-binding-interacting homolog (RIN1)	AALBA5B827346T1
3120	aalba5_s00046057:16,198	309 bp downstream	Sorbitol dehydrogenase (DHSO)	AALBA5B083042T1
3313	aalba5_s00049423:3040	909 bp upstream	Translation initiation factor (SDA1)	AALBA5B666590T1
3870	aalba5_s00060613:17,756	21,499 bp upstream	Ribonuclease HI (RNH)	AALBA5B308154T1
3874	aalba5_s00060695:20,797	21,154 bp upstream	UDP-glucose 4-epimerase (GALE2)	AALBA5B999424T1

**Table 7:** Molecular markers (SNPs) associated with phenotypic traits found within or close to an annotated gene (“Position”) in *A. alba*, specifying their exact location in the genome, as well as the protein encoded by such genes.

SNP	Location	Position	Protein	ID
1	aalba5_s00000025:18,4813	Within the gene	Probable kinase (Y1960)	AALBA5B157798T1
259	aalba5_s00002236:37,636	6142 bp upstream	Farnesyl pyrophosphate synthase (FPPS)	AALBA5B673769T1
887	aalba5_s00009946:20,716	22 bp upstream	Zinc finger 10 (ZFP10)	AALBA5B909389T1
1769	aalba5_s00022666:63,643	55,996 bp downstream	Expansin (EXPA8)	AALBA5B425104T1
2319	aalba5_s00031591:9355	Within the gene	RecQ-mediated genome instability protein (RMI-1)	AALBA5B1147052T1
3101	aalba5_s00045724:48,524	4919 bp downstream	Ribonuclease H (RNHX1)	AALBA5B088054T1
3238	aalba5_s00048104:19,924	284 bp upstream	SUN domain-containing protein (SUN1)	AALBA5B001978T1
3878	aalba5_s00060735:27,446	16,784 bp downstream	Aluminum-activated malate transporter (ALMTE)	AALBA5B787661T1

**Table 8:** Brief characterization of the study sites, including their geographical position and climate information. Altitude was measured in meters above sea level, mean temperature (T) was measured in °C, and annual precipitation (P) was measured in mm.

Populations	Cotatuero (CO)	Gamueta (GA)	Paco Ezpela (PE)	Paco Mayor (PM)	Selva de Oza (SO)
Latitude	42.65	42.88	42.74	42.71	42.84
Longitude	−0.04	−0.79	−0.83	−0.64	−0.70
Altitude	1456	1450	1116	1322	1220
Mean T	5.22	6.23	9.74	8.55	7.47
Annual P	1167	1120	867	944	1028

**Table 9:** Bioclimatic variables for each *A. alba* population’s location. BIO1: annual mean temperature (°C). BIO2: mean diurnal range (°C). BIO3: isothermality (%). BIO12: annual precipitation (mm). BIO15: precipitation seasonality (%).

Variable	CO	GA	PE	PM	SO
BIO1	5.22	6.23	9.74	8.56	7.47
BIO2	10.1	9.48	9.41	9.78	9.53
BIO3	36.62	35.75	37.33	37.48	36.35
BIO12	1167	1120	867	944	1028
BIO15	19.30	21.11	21.87	21.06	20.79

**Table 10:** Abiotic soil variables for each *A. alba* population (mean value ± standard error): percentage of nitrogen (% N), carbon-to-nitrogen ratio (C/N), percentage of organic carbon (% org C), assimilable phosphorus (P); percentage of clay (% clay), percentage of silt (% silt), percentage of sand (% sand), and Saxton value (Saxton).

Variable	CO	GA	PE	PM	SO
% N	0.87 ± 0.10	0.34 ± 0.02	0.37 ± 0.01	0.32 ± 0.02	0.32 ± 0.01
C/N	18.93 ± 0.36	17.46 ± 0.39	18.32 ± 0.38	15.93 ± 0.41	14.04 ± 0.44
% org C	16.44 ± 1.79	5.97 ± 0.45	6.87 ± 0.33	5.11 ± 0.30	4.53 ± 0.30
P (ppm)	66.98 ± 7.02	18.36 ± 1.62	13.50 ± 1.28	12.05 ± 1.83	8.16 ± 0.87
% clay	6.89 ± 0.45	15.69 ± 0.56	21.74 ± 0.61	25.36 ± 0.86	24.88 ± 0.55
% silt	29.91 ± 0.72	42.36 ± 0.84	40.70 ± 0.67	43.90 ± 0.85	50.65 ± 0.72
% sand	63.20 ± 1.07	41.95 ± 1.24	37.56 ± 1.21	30.74 ± 1.49	24.47 ± 1.08
Saxton	2.11 ± 0.05	1.63 ± 0.04	1.02 ± 0.04	0.85 ± 0.07	1.23 ± 0.05

**Table 11:** Biotic soil variables for each *A. alba* population (mean values ± standard error in nmoles): general phospholipid fatty acid (FAME) content, eukaryote biomass, Gram-negative bacteria biomass, Gram-positive bacteria biomass, Actinomycetes biomass, fungi biomass, arbuscular mycorrhizal (AM) fungi biomass, and anaerobic bacteria biomass.

Variable	CO	GA	PE	PM	SO
General FAME	63.57 ± 4.12	41.91 ± 3.00	29.96 ± 1.16	24.86 ± 1.72	30.63 ± 1.96
Eukaryote	7.99 ± 0.91	4.45 ± 0.47	6.31 ± 0.32	5.13 ± 0.68	4.91 ± 0.89
Gram-negative	181.97 ± 10.71	116.56 ± 9.22	113.75 ± 3.43	90.33 ± 5.63	123.61 ± 8.62
Gram-positive	98.25 ± 5.30	70.02 ± 4.39	74.67 ± 2.04	64.81 ± 3.88	78.15 ± 4.75
Actinomycetes	36.34 ± 2.67	20.82 ± 1.36	24.93 ± 1.09	20.21 ± 1.27	23.85 ± 1.42
Fungi	15.51 ± 1.30	11.36 ± 1.47	16.10 ± 1.39	15.58 ± 1.82	11.87 ± 1.84
AM Fungi	13.95 ± 0.90	7.49 ± 0.68	9.80 ± 0.39	8.62 ± 0.59	10.63 ± 0.89
Anaerobe	3.22 ± 0.27	1.76 ± 0.11	1.81 ± 0.07	1.64 ± 0.11	1.88 ± 0.14

**Table 12:** Description of functional dendrophenotypic traits related to tree-level growth patterns, which were based on the basal area increment (BAI). See [15,38] for details.

Trait	Description
BAI5, BAI10, BAI20	Mean BAI in the last 5, 10, and 20 years, respectively (cm^2^)
BAI mean 1979–1999, BAI mean 1999–2019	Mean BAI for the indicated time span (cm^2^)
BAI trend 1979–1999, BAI trend 1999–2019	Slope of the BAI for the indicated time span (cm^2^ year^−1^)
Within tree BAI autocorr. 1979–1999, Within tree BAI autocorr. 1999–2019	Tree-level first-order Pearson’s autocorrelation of the BAI over time for the indicated time span
Among trees BAI intercorr. 1979–1999, Among trees BAI intercorr. 1999–2019	Pearson’s correlation between the tree-level BAI and population mean BAI for the indicated time span
CV 1979–1999, CV 1999–2019	Tree-level coefficient of variation of the BAI (standard deviation/mean 100) for the indicated time span (%)

**Table 13:** Description of functional dendrophenotypic traits related to tree-level climate sensitivity. They represent the Pearson’s correlation coefficient of the basal area increment (BAI) and the described climate variable for the time span from 1961 to 2019. SPEI stands for Standardized Precipitation Evapotranspiration Index. See [15,38] for details.

Trait	Description
PsuP, PauP	Previous summer and autumn precipitation, respectively
Pwi, Psp, Psu, Pau, Pye	Winter, spring, summer, autumn, and year precipitation, respectively
TsuP, TauP	Previous summer and autumn temperature, respectively
Twi, Tsp, Tsu, Tau, Tye	Winter, spring, summer, autumn, and year temperature, respectively
spei_sup, spei_aup	Previous summer SPEI, previous autumn SPEI
spei_wi, spei_sp, spei_su, spei_au, spei_ye	Winter, spring, summer, autumn, and year SPEI, respectively

## Data Availability

Raw genomic sequences can be accessed at NCBI’s Sequence Read Archive (SRA), under the BioProject with accession number PRJNA947483.

## Supplementary Materials

The following supporting information can be downloaded at: https://www.mdpi.com/article/10.3390/plants12142607/s1, Figure S1: Detection of selection signatures considering all populations. SNPs to the right of the vertical line show significant signatures of selection (*q*-value < 0.05).; Table S1: Original number of samples from each sampling point and final number of retained samples after filtering; Table S2: Analysis of molecular variance (AMOVA); Table S3: Genome–environment associations (GEA) with bioclimatic variables. SNPs with selection signatures are marked in bold; Table S4: Genome–environment associations (GEA) with abiotic soil variables. SNPs with selection signatures are marked in bold; Table S5: Genome–environment associations (GEA) with biotic soil variables. SNPs with selection signatures are marked in bold; Table S6: Genotype–phenotype associations; Table S7: Tree-level values of functional traits related to leaf characteristics; Table S8: Tree-level values of dendrophenotypic traits related to growth patterns; Table S9: Tree-level values of dendrophenotypic traits related to climate sensitivity; Table S10: Tree-level values of dendrophenotypic traits related to responses to documented drought periods.
