# Supplementary material for: Climate and Soil Microsite Conditions Determine Local Adaptation in Declining Silver Fir Forests
Source: Plants (Basel). 2023 Jul 10;12(14):2607. doi: 10.3390/plants12142607 (PMC10384727; doi:10.3390/plants12142607)
Supplement: Supplementary file 1 [file plants-12-02607-s001.zip › plants-2430301-supplementary.pdf]

Supplementary materials

1. *Figures*

Figure S1. Detection of selection signatures considering all populations. SNPs to the right of the vertical line show significant signatures of selection (*q*-value < 0.05).

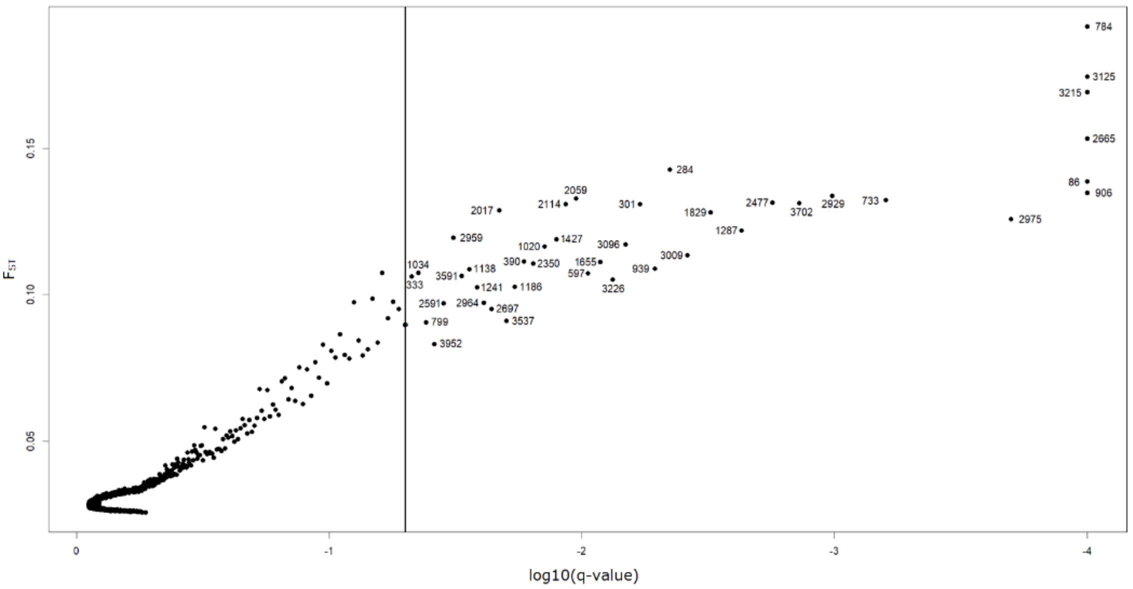

2. *Tables*

Table S1. Original number of samples from each sampling point and final number of retained samples after filtering.

Population		Sequenced Samples	Retained Samples
**CO**	Non-declining	15	15
	Declining	15	13
	Saplings	21	19
**GA**	Non-declining	15	14
	Young	5	4
**PE**	Non-declining	20	20
	Declining	18	18
	Saplings	39	36
**PM**	Non-declining	19	19
	Declining	20	20
	Saplings	39	38
**SO**	Non-declining	20	18
	Saplings	20	20

Table S2. Analysis of molecular variance (AMOVA).

Source of Variance	F-Stat	F-Value	% of Total Variance	*p*-Value
Within individuals	F_{IT}	0.123	87.7	-
Among individuals	F_{IS}	0.099	9.7	0.000
Among populations	F_{ST}	0.026	2.6	0.000

Table S3. Genome-environment associations (GEA) with bioclimatic variables. SNPs with selection signatures are marked in bold.

SNP	BIO1	BIO2	BIO3	BIO12	BIO15
159	X	X		X	X
179					X
278					X
350	X			X	
448		X			
608		X			
681					X
752		X			X
**784**		X			
811					X
858		X			
**939**		X			
948		X			X
983	X	X			X
1172					X
1188	X		X	X	
1344					X
1517		X			X
1544	X			X	
1808		X			
1934					X
2061					X
2119		X			
2128		X			
2131					X
2249					X
2438					X
2452		X			
2459	X				
2484	X				
**2591**	X			X	X
2645		X			
2794		X			
2940					X
2968		X			
2981					X
3063	X			X	
3111	X			X	X
3306	X			X	
3313					X
3337					X
3380					X
3400	X			X	
3450	X				
3491	X			X	
**3537**	X	X		X	X

3779	X			X	
Total	16	18	1	12	24

Table S4. Genome-environment associations (GEA) with abiotic soil variables. SNPs with selection signatures are marked in bold.

SNP	%N	C/N	% Organic C	P (ppm)	% Clay	% Silt	% Sand	Saxton
197					X			X
238	X		X	X				X
287					X			
368							X	
764					X			X
900		X						
948	X		X	X				
971		X			X		X	X
983				X	X		X	X
1074					X		X	
**1287**								X
1592	X							
1673	X		X	X				
1808	X							
**1829**		X			X		X	
2131					X			X
2392								X
2587				X	X		X	X
**2665**					X		X	X
2775								X
2794				X				
2814				X				
2835	X		X	X	X		X	
2928						X		
3000		X						
3120	X							
3226	X			X				X
3286		X						
3313	X		X	X	X		X	X
3380								X
**3537**	X		X	X	X	X	X	X
3742	X		X					
3870					X			X
3874	X		X	X				
**3952**								X
Total	12	5	8	12	14	2	10	17

Table S5. Genome-environment associations (GEA) with biotic soil variables. SNPs with selection signatures are marked in bold.

SNP	General FAME	Eukaryote	Gram Negative	Gram Positive	Actinomycetes	Fungi	AM Fungi	Anaerobe
159			X	X				X
197	X		X	X				X
238	X		X	X	X			X
288			X	X	X		X	
434				X				
681			X					
764	X							
948		X	X	X	X			X
971	X							
983	X							
1074	X							
1425			X					
1555				X	X		X	
1673								X
1712			X	X				
1756						X		
1808				X	X			X
2131	X							
2587	X							
**2591**								X
**2665**	X			X	X			
2835	X							
2997				X				
3049							X	
3104				X				
3120		X			X			X
**3226**	X		X	X	X		X	X
3313	X		X	X	X			X
3380			X	X	X			X
**3537**	X		X	X	X		X	X
3742		X						X
3870	X							
3874		X	X	X	X		X	X
Total	14	4	13	17	12	1	6	14

Table S6. Genotype-phenotype associations.

SNP	LMA	LDMC	Rc86	Rt98	Rs98	Rt0506	Rc0506	Rs0506	Rs12
1				X	X				
259	X								
797		X							
887						X			
1086							X		
1104					X				
1277					X				
1669	X								
1769								X	
2270			X						
2319					X				
2431	X								
2797					X				
2882		X							
3101									X
3205		X							
3238				X	X				
3718									X
3878					X				
Total	3	3	1	2	7	1	1	1	2

Table S7. Tree-level values of functional traits related to leaf characteristics. Available at: https://github.com/igargar/Plants_supplementary_excel/blob/main/Supplementary%20tables%20S7-S10.xlsx.

Table S8. Tree-level values of dendrophenotypic traits related to growth-patterns. Available at: https://github.com/igargar/Plants_supplementary_excel/blob/main/Supplementary%20tables%20S7-S10.xlsx.

Table S9. Tree-level values of dendrophenotypic traits related to climate sensitivity. Available at: https://github.com/igargar/Plants_supplementary_excel/blob/main/Supplementary%20tables%20S7-S10.xlsx.

Table S10. Tree-level values of dendrophenotypic traits related to response to documented drought periods. Available at: https://github.com/igargar/Plants_supplementary_excel/blob/main/Supplementary%20tables%20S7-S10.xlsx.
